# The Association between Levels of Trust in the Healthcare System and Influenza Vaccine Hesitancy among College Students in Israel

**DOI:** 10.3390/vaccines11111728

**Published:** 2023-11-19

**Authors:** Keren Dopelt, Anuar Abudin, Sophie Yukther, Tatyana Shmukler, Nadav Davidovitch

**Affiliations:** 1 Department of Public Health, Ashkelon Academic College, Ashkelon 78211, Israel, sophieyu16@edu.aac.ac.il (S.Y.),; 2 School of Public Health, Faculty of Health Sciences, Ben Gurion University of the Negev, Beer Sheva 84105, Israel; nadavd@bgu.ac.il

**Keywords:** trust in the healthcare system, influenza, vaccines, vaccine hesitancy, students, Israel

## Abstract

Influenza is a contagious respiratory disease caused by the influenza virus. Vaccination proves an effective approach to preventing influenza and minimizing the risk of experiencing associated complications. However, the influenza vaccine coverage rate among Israeli college students is low due to a sense of complacency, lack of knowledge, and vaccine hesitancy. The current study examined the relationship between the level of trust in the healthcare system and influenza vaccine hesitancy among college students in Israel. This cross-sectional study was conducted via an online questionnaire in April–May 2023. In total, 610 students were surveyed, of whom 57% had been vaccinated against influenza in the past; however, only 12% were vaccinated this year. Negative, significant, and moderate relationships were found between the level of trust in the healthcare system and influenza vaccine hesitancy. Students who had been vaccinated in the past had a higher level of trust in the healthcare system and a lower level of vaccination hesitancy. The linear regression model revealed that the variables of being a woman, not Jewish, vaccinated, and trusting the Ministry of Health, family doctor, and health professionals were associated with a decrease in vaccine hesitancy. These findings are in line with previous research in the field. Based on the present results, it may be advisable to develop intervention programs aimed at increasing confidence in the healthcare system and vaccinations by providing knowledge and addressing students’ concerns regarding vaccination.

## 1. Introduction

Vaccine hesitancy refers to delay in accepting or outright refusal of vaccines, even when vaccination services are readily available [1]. This issue has been recognized by the World Health Organization [2] as a major health concern, and vaccine hesitancy is listed among the top ten threats to public health. Influenza infections result in approximately 3–5 million cases of severe illness and 290,000–650,000 respiratory-related deaths worldwide each year [3,4]. Influenza vaccination is one of the most efficient approaches to reducing the health, societal, and economic impacts of influenza [5,6]. The Israeli Ministry of Health recommends receiving an influenza vaccine for every individual above six months old, with an emphasis on the children and the elderly population. The vaccines are provided free of charge at clinics distributed in every neighborhood in Israel, ensuring very high accessibility to vaccines. Despite the seriousness of this illness and the availability of safe vaccines, influenza vaccination rates continue to be low. This presents a global challenge and adds to the burden that this disease imposes on healthcare systems around the world [7]. The healthcare system plays an essential role in encouraging vaccine uptake for influenza. Influenza vaccination is crucial for the general population, including student populations in close contact in classrooms and other social settings. Studies have reported low seasonal influenza vaccination rates among students, with coverage ranging from 12% to 30% [8]. If the student population is not vaccinated against influenza, the global population will not meet the World Health Organization (WHO) aim for approximately 75% coverage of influenza vaccination. While global healthcare systems face the need to address vaccine hesitancy among the general public, particular emphasis needs to be placed on university students in this regard. Influenza symptoms may persist for multiple weeks, impacting students’ class attendance, academic achievements, social engagements, and productivity [9]. Additionally, influenza transmission rates within university settings can be notably elevated due to the concentration of dozens of students in shared spaces [10]. Moreover, an influenza outbreak on campus holds the potential to extend its spread to the broader community surrounding students, encompassing friends, family members, and high-risk population groups [11].

Previous studies have explored trust in the healthcare system and trust in healthcare providers when seeking to explain health-related behavior. These analyses have revealed a positive correlation between trust in physicians and adherence to medical recommendations, thereby leading to improved health outcomes [12]. Conversely, lower levels of trust are linked to reduced utilization of preventive health screenings and lower uptake of the influenza vaccine [13,14,15]. The SAGE Working Group on Vaccine Hesitancy recognized trust in the healthcare system and healthcare providers as pivotal determinants of vaccine hesitancy [1,16]. Research has indicated higher levels of vaccine hesitancy regarding influenza, COVID-19, or HPV vaccines among specific demographic groups compared to the general population. These groups include healthcare workers, minority communities, and individuals with lower socioeconomic status [17,18,19]. Research has underscored the significant impact that a doctor’s recommendation can have on a patient’s inclination to receive vaccinations [20,21,22]. Conversely, individuals who opt not to get vaccinated often cite a lack of trust in these institutions as a primary reason for refusing vaccines [23]. Groups with diminished trust in the public health system are approximately half as likely to receive vaccinations compared to those with elevated levels of trust [24] (Gilles et al., 2011). Moreover, healthcare professionals who themselves are hesitant about vaccinations may not adequately address their patients’ vaccine concerns [25].

Trust in the public health organizations and experts who provide vaccine recommendations is a significant factor influencing individuals’ decisions and beliefs regarding vaccines [23,26]. The literature suggests that trust in the healthcare system is built on healthcare professionals’ competence (skills and knowledge) and how the healthcare system and its actors (medical staff) work to benefit the patient through acting with integrity, maintaining individual privacy and medical confidentiality, and showing empathy and respect [27]. A healthcare system based on trust contributes to creating broader social value, based on the premise that the healthcare system not only produces healthy outcomes among the public and prioritizes improving the state of health in society but, as a social institution, also establishes social norms shaping human behavior [28]. In recent years, Israelis have exhibited relatively low levels of public trust in the healthcare system compared to other countries in the OECD, with only half of the Israeli public (52%) reporting that they believed that they would receive the best treatment for a severe illness [29].

Low influenza vaccination rates among students are a worldwide occurrence [30]. While vaccine hesitancy has been extensively researched in the general adult population, young individuals have not been a strategic focus of vaccination encouragement and public health communication efforts from the perspective of the Israeli Health and Public Health system. In general, students are young and tend to perceive themselves as healthy with a low risk of falling ill despite the rapidity with which influenza can spread through campuses. Given these concerns regarding the reluctance of students to be vaccinated, in this study, we sought to explore their level of trust in the healthcare system and whether this trust is associated with influenza vaccine hesitancy. The findings help understand the level of trust in the healthcare system among students in Israel and its connection to influenza vaccination hesitancy, aiding in the development of intervention programs accordingly.

## 2. Materials and Methods

### 2.1. Research Procedure

This descriptive, cross-sectional study was undertaken with students from Ashkelon Academic College. In 2023, approximately 4200 students studied at this college in the academic track. Approval for this study was obtained from Ashkelon Academic College Ethics Committee (approval #42-2023). Data were obtained from all College departments. The study ran from 2 April 2023 to 12 May 2023, concomitant with the end of the influenza vaccination season in Israel. The survey questionnaire was programmed using Qualtrics (Qualtrics, Provo, UT, USA) and was distributed to all students via email. One reminder to fill out the questionnaire was sent via email three weeks following its initial distribution. A total of 703 students responded, with 610 completing at least 90% of the questionnaire. This represented a response rate of 87% of all respondents and 15% of the research population. On average, it took 5 ± 1.44 min to complete the questionnaire. The introductory page of the questionnaire explained the aims of this study and ensured anonymity. Completing the questionnaire indicated the students’ voluntary agreement and informed consent to participate. Students could stop responding at any time, and there was no obligation to answer any specific question.

### 2.2. Tools

We used an online, closed, anonymous, self-completed questionnaire to collect the data for this study. A professional translator translated the questionnaire from English into Hebrew. The Hebrew-translated questionnaire was then administered to 10 students not attending Ashkelon Academic College to verify the comprehensibility of the questions. The questionnaire was revised based on their feedback. Moreover, the questionnaire underwent content validation through assessment by an expert in public health and epidemiology and an expert in infectious diseases.

The final questionnaire comprised the following components:

1. Demographic information: Gender, age, marital status, religion, department, and year of study.
2. Vaccination history: This included questions drawn from Ryan et al. [11]: Have you ever been vaccinated against the flu? Have you been vaccinated against influenza this year?
3. Vaccine hesitancy: This included six questions from Silva et al. [31]. The respondents were asked to indicate their degree of agreement with each statement in the questionnaire on a Likert scale ranging from 1 (not at all) to 5 (strongly agree) with the option to answer “don’t know”. The average of the answers was calculated for each participant after reversing the scales for questions 1 and 6 and dropping the “don’t know” answers. A higher score was indicative of higher levels of vaccine hesitancy. Cronbach’s α for reliability was 0.77.
4. Level of trust in the healthcare system: This included three questions from Jennings et al. [32] measuring the level of trust in one’s doctor, the Ministry of Health, and medical professionals. The response scale ranged from 1 (not at all) to 5 (strongly agree). The variable was constructed by calculating the mean response for each participant. The mean ranged from 1 to 5, with a higher score indicating a higher level of trust in the healthcare system. Cronbach’s α for reliability was 0.82.

### 2.3. Data Analysis

The data were analyzed using SPSS 29.0 (IBM, Armonk, NY, USA). Relationships between the variables were examined using Pearson correlation analyses. Since the variables met the criteria of normal distribution, differences between student groups were assessed utilizing *t*-tests for independent samples and one-way analyses of variance (ANOVAs). To predict the extent of vaccination hesitancy, a multiple linear regression model was used. The model included variables that have been found to be associated with the dependent variable in the univariate analyses. Significance in reported *p*-values relied on two-sided tests and were considered significant when they fell below 0.05.

## 3. Results

### 3.1. Participant Characteristics and Influenza Vaccination History

In total, 610 students participated in this study, of whom 60% were women, 53% were in relationships, and 21% had children. Most participants were Jewish (83%). Nearly half studied in the Faculty of Social Sciences (46%), 35% in Health Sciences, and 19% in Computer Science and Management. The mean age of the respondents was 27.64 ± 7.20 years. The survey population resembled the college’s population in terms of gender, age, and faculties composition. More than half had been vaccinated in the past (57%; 61% when excluding participants who could not remember). Among these participants, 12% were vaccinated, 44% intended to get vaccinated, 8% were undecided, and 36% did not intend to get vaccinated. No significant differences were found between the faculties with respect to vaccination history. However, significant differences between faculties were detected regarding vaccination in the study year (χ^2^ = 24.66, *p* < 0.001), with more students in Health Sciences having been vaccinated or intending to be vaccinated (16% and 47%, respectively) compared to Computer Science and Management students (14% and 52%, respectively) or Social Sciences students (11% and 35%, respectively). The characteristics of these participants and their influenza vaccination history are summarized in Table 1.

### 3.2. Level of Trust in the Healthcare System

The distribution of responses to statements that examined the level of trust in the healthcare system is presented in Table 2 after combining categories as follows: answers 1 and 2 were incorporated into the category “weakly agree”, while answer 3 was classified as “moderately agree”, and answers 4 and 5 were integrated into the category “strongly agree”.

To assess the level of trust in the healthcare system variable, the mean response for each participant was calculated, with a computed value of 3.06 (SD = 0.88).

### 3.3. Influenza Vaccine Hesitancy

The distribution of responses to statements that examined influenza vaccine hesitancy is presented in Table 3 after combining categories as follows: answers 1 and 2 were combined into the category “weakly agree”, answer 3 remained “moderately agree”, and answers 4 and 5 were integrated into the category “strongly agree”.

For the purposes of constructing the influenza vaccine hesitancy variable, we calculated the mean response for each participant when excluding the “I don’t know” responses and reversing the scale for questions 1 and 6, yielding a mean value of 3.11 (SD = 0.70).

### 3.4. Relationships between the Level of Trust in the Healthcare System and Influenza Vaccine Hesitancy

Negative, significant, and moderate relationships were found between the level of trust in the Ministry of Health, one’s family doctor, health professionals, general trust in the healthcare system, and influenza vaccine hesitancy (r*_p_* = −0.45, *p* < 0.001; r*_p_* = −0.21, *p* < 0.001; r*_p_* = −0.44, *p* < 0.001; r*_p_* = −0.43, *p* < 0.001, respectively). In other words, the higher the level of trust in the healthcare system, the lower the degree of influenza vaccine hesitancy.

### 3.5. The Relationship between Influenza Vaccination History and the Study Variables

Significant differences were found between students who had been vaccinated in the past and students who had not been vaccinated with respect to their levels of trust in the healthcare system (t = 3.89, *p* < 0.001) and vaccination hesitancy (t = 6.69, *p* < 0.001). Specifically, students who had been vaccinated in the past exhibited a higher level of trust in the healthcare system than unvaccinated students (3.17 vs. 2.87, respectively) and a lower level of vaccination hesitancy (2.95 vs. 3.23, respectively).

### 3.6. Differences between Faculties

Significant differences were found between faculties in terms of level of trust in the healthcare system (F_(543)_ = 4.46, *p* < 0.05). Students in the Health Sciences faculty demonstrated the highest level of trust, followed by students in Social Sciences and, finally, students in Computer Science and Management (averages of 3.22, 3.01, and 2.92, respectively). Scheffe post-hoc tests revealed that students in the Health Sciences faculty had significantly higher knowledge levels than students in the two other faculties.

Furthermore, significant differences were found between the faculties with respect to levels of influenza vaccine hesitancy (F_(565)_ = 3.17, *p* < 0.05). Computer Science and Management students had the highest hesitancy level, followed by students in Social Sciences and, finally, Health Sciences (averages of 3.22, 3.10, and 3.00, respectively). Scheffe post-hoc tests revealed that students in the Faculty of Computer Science and Management exhibited significantly higher hesitancy levels than Health Science students.

### 3.7. Regression Model to Predict Influenza Vaccine Hesitancy

Table 4 presents the results of a linear regression model predicting influenza vaccine hesitancy. The coefficients and *p*-values shed light on how each variable predicts vaccine hesitancy. Being female, not Jewish, vaccinated, and trusting the Ministry of Health, the family doctor, and health professionals were all found to be associated with lower vaccine hesitancy. The best predictors of this lower vaccine hesitancy were the level of trust in the Ministry of Health, the level of trust in health professionals’ recommendations, and the incidence of being vaccinated in the past. The explained variance of the model was 30% (*p* < 0.001).

## 4. Discussion

Our results revealed that trust in the Ministry of Health and the belief that it works for the benefit of the entire population of Israel is low (average 2.67) among the college’s students, while levels of trust in the recommendations of health professionals regarding vaccines are higher but not satisfactory (average 2.98). Nevertheless, study participants were found to generally trust their family doctor’s recommendations (average 3.55). Previous studies conducted in Western countries have also highlighted the disparity in trust and satisfaction levels between local health services and the national healthcare system. While trust and satisfaction rates often range from 80 to 90% at the local level, they decline to approximately 50–60% at the national level. This emphasizes the greater trust that individuals have in their local doctors compared to the national level [33,34,35].

Negative, significant, and moderate relationships were found between all the dimensions of trust in the healthcare system and influenza vaccine hesitancy. The literature indicates that public trust in healthcare professionals is crucial for the health system to function efficiently. Trust is the primary factor influencing individuals’ vaccination decisions [21,36]. Among other things, when making decisions, individuals must trust the information they are being provided [37]. In the context of vaccinations, decision-making is associated with trust in government and public health professionals [26]. In line with our findings, studies have reported a negative correlation between an individual’s vaccine hesitancy and their trust in the healthcare system and healthcare workers [38,39,40]. Physicians’ advocacy of vaccinations is recognized as one of the most influential factors affecting public attitudes toward vaccinations [20,21,22]. Conversely, hesitancy and skepticism regarding vaccinations can be linked, in part, to a diminished level of trust in physicians [23,41].

A cross-national study conducted during the COVID-19 pandemic found that when trust levels in the healthcare system and the WHO were higher, vaccine hesitancy levels were lower [42]. A similar study conducted at the University of North Carolina found that as students’ levels of trust in the healthcare system and other information sources rose, their hesitancy levels declined [43]. A survey distributed among students from the Central University Center of Baia Mare (Romania) observed a significant correlation between high levels of trust in institutions and the intention to vaccinate [44]. The link between trust in the healthcare system, attitudes towards vaccines, and vaccine hesitancy can also be explained using the health belief model [45]. According to this model, in order for a change to be effected in a person’s behavior or, in this case, to induce a shift from vaccine hesitancy to vaccine acceptance, the person must believe and have confidence that the action being taken can indeed benefit them, meaning that, in this case, the vaccine can help them. The more a given individual trusts the system, the more likely they are to believe that the vaccine can benefit them.

The present results indicated that students who have been previously vaccinated exhibit higher levels of trust in the healthcare system and lower levels of hesitancy compared to students who have not been vaccinated. The theory of planned behavior [46] argues that attitudes and social norms influence the behavior of a given individual. In other words, those who have already been vaccinated likely hold more positive attitudes such that they are less hesitant to vaccinate again. Additionally, it can be assumed that individuals who have been vaccinated live in an environment where social norms emphasize trust in the healthcare system and vaccines.

We also found that students from the Faculty of Health Sciences have the highest level of trust and the lowest levels of vaccine hesitancy level compared to students from other disciplines. Similar findings were also obtained in a study conducted at a university in Saudi Arabia [47] and in Japan [48]. Generally, health science students learn about the healthcare system in greater depth than students from other disciplines and encounter it during their internships. This results in higher levels of trust in this system among them compared to students who come into contact with the health system only as patients. Health science students also learn more about the mechanism of vaccines, and this knowledge reduces vaccine hesitancy.

The linear regression model revealed an association between decreased vaccine hesitancy and the variables of being a woman, not Jewish, vaccinated, and trusting the Ministry of Health, family doctor, and health professionals. A study by Shon et al. [49] found that more female students were vaccinated than male students, suggesting that among students, males exhibit higher levels of vaccine hesitancy, as was found in the current study. Also consistent with the results of the current study’s regression analysis are the findings of other studies indicating that previously vaccinated students exhibit less vaccine hesitancy [11,49,50]. With respect to religion, the current study’s findings align with those from other studies, indicating that the Arab sector in Israel has less trust in state institutions, including the healthcare system [21,51].

When delving into the association between trust and vaccine hesitancy, it is crucial to acknowledge the erosion of public trust in governments, healthcare systems, and experts on a global scale due to the influence of the COVID-19 pandemic [40]. The pandemic unleashed a flood of misinformation, famously termed an “Infodemic” [52], contributing to the rise in vaccine hesitancy. Freiman [40] advocates for mitigating vaccine concerns and fostering trust among the hesitant by actively engaging and imparting knowledge [53]. It is reasonable to anticipate that improving trust will streamline intricate decisions about vaccination [54].

### Study Limitations

The present research effort was limited to students from a single college, potentially affecting the ability to generalize these findings to students nationwide. Furthermore, most participants had not been vaccinated against influenza in the study year, and a significant portion expressed no intention of becoming vaccinated. This suggests a potential selection bias, wherein students with greater vaccine hesitancy may have been more inclined to participate in the survey.

## 5. Conclusions

Trust in the Ministry of Health, family doctors, and public health professionals are important predictors of vaccine hesitancy. Physicians may be able to build on the trust their patients have in them to address vaccine concerns and increase vaccination rates against influenza. To persuade students to vaccinate, interventions centered on transferring professional knowledge and allaying concerns about vaccinations can be conducted on campuses in collaboration with the management of these institutions, the Ministry of Health, and doctors from nearby hospitals or clinics. It is crucial to make it clear to students that young people can also become seriously ill with influenza and that they are at high risk of infection due to overcrowding in classrooms and other social settings. Lastly, steps to build trust between various components of the healthcare system and the student population should be taken, viewing these students as ambassadors for improving vaccination rates.

## Figures and Tables

**Table 1:** The characteristics and influenza vaccination history of study participants.

Characteristics	*n*	%
Gender		
Male	243	40
Female	367	60
In relationship	324	53
Have children	128	21
Jewish	509	83
Faculty		
Health Sciences	202	35
Social Sciences	262	46
Computers Science and Management	106	19
Year of studies		
1st	310	51
2nd	198	32
3rd and 4th	102	17
Vaccinated against influenza in the past		
Yes	351	57
No	223	37
Do not remember	36	6
Vaccinated this year against influenza:		
Yes	76	12
Intend to vaccinate	269	44
Do not intend to vaccinate	217	36
Undecided	48	8

**Table 2:** The distribution of responses to the questionnaire focused on the level of trust in the healthcare system questionnaire.

Statement	Weakly (%)	Moderately (%)	Strongly (%)	Mean ± SD
I trust the Ministry of Health, which works for the benefit of the entire population	46	32	22	2.67 ± 1.07
I trust my family doctor’s recommendations	13	30	57	3.55 ± 0.93
I trust the recommendations of the health professionals regarding vaccines	32	33	35	2.98 ± 1.08

**Table 3:** Distribution of responses to the influenza vaccine hesitancy questionnaire.

Statement	Weakly (%)	Moderately (%)	Strongly (%)	Don’t Know (%)	Mean ± SD ^1^
I am (not) worried about getting influenza *	40	19	36	5	3.01 ± 1.36
I am concerned about the ineffectiveness of the influenza vaccine	40	9	56	5	3.59 ± 1.61
I am concerned about the limited information available about the influenza vaccine	28	10	58	4	3.63 ± 1.62
I will only get the influenza shot if it becomes mandatory	70	10	13	7	1.84 ± 1.28
I think the influenza shot is not safe	29	13	50	9	3.30 ± 1.52
I (do not) recommend family/friends to get vaccinated against influenza *	28	24	30	18	3.09 ± 1.37

^1^ The mean was calculated without including the “I don’t know” option. * Opposite questions. The data are presented in reverse rank order.

**Table 4:** Linear regression model results for predicting influenza vaccine hesitancy.

Variable	B	β	*p*
Gender (0—male; 1—female)	−0.21	−0.12	0.001
Religion (0—Jewish; 1—not Jewish)	0.20	0.09	0.020
Vaccinated (0—no; 1—yes)	−0.28	−0.16	<0.001
Trust in the Ministry of Health	−0.23	−0.29	<0.001
Trust in the family doctor	−0.10	−0.11	0.019
Trust in health professionals	−0.22	−0.28	<0.001
Adjusted R Square	0.30, *p* < 0.001		
F	39.43, *p* < 0.001		
N	545		

## Data Availability

The data presented in this study are available on request from the corresponding author.
